# (2*E*)-1-Phenyl-2-[1-(2-phenyl­prop-2-en-1-yl)pyrrolidin-2-yl­idene]ethanone

**DOI:** 10.1107/S1600536812044443

**Published:** 2012-11-03

**Authors:** Lee G. Madeley, Garreth L. Morgans, Andreas Lemmerer, Joseph P. Michael

**Affiliations:** a Molecular Sciences Institute, School of Chemistry, University of the Witwatersrand, Private Bag 3, PO WITS, 2050, Johannesburg, South Africa

## Abstract

The title compound, C_21_H_21_NO, is a vinyl­ogous amide (enaminone) produced by reaction of 1-(2-phenyl­prop-2-en-1-yl)pyrrolidine-2-thione with phenacyl bromide. In the mol­ecule, the phenyl rings are twisted from the mean plane of the pyrrolidine ring by 11.2 (1) and 67.3 (1)°. In the crystal, weak C—H⋯O hydrogen bonds link the mol­ecules related by translation along the *b* axis into chains.

## Related literature

For details of the synthesis of enamino­nes, see: Roth *et al.* (1971 ▶). For applications of enamino­nes in alkaloid synthesis, see: Michael *et al.* (1999 ▶). For a related enaminone structure, see: Lemmerer *et al.* (2007 ▶).
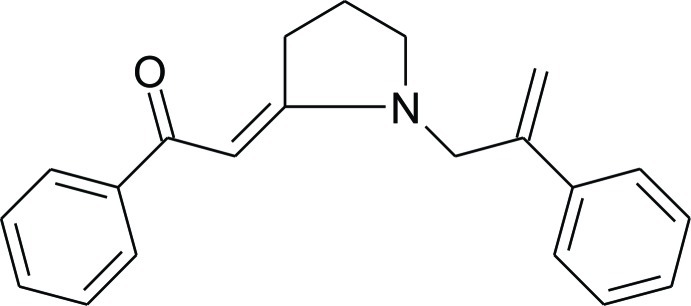


## Experimental

### 

#### Crystal data

C_21_H_21_NO
*M* *_r_* = 303.39
Triclinic, 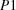

*a* = 5.7806 (6) Å
*b* = 7.9407 (7) Å
*c* = 9.6089 (9) Å
α = 82.579 (7)°
β = 76.793 (7)°
γ = 83.510 (7)°
*V* = 424.21 (7) Å^3^
*Z* = 1
Mo *K*α radiation
μ = 0.07 mm^−1^
*T* = 293 K
0.4 × 0.2 × 0.19 mm

#### Data collection

Bruker APEXII CCD area-detector diffractometer
5222 measured reflections
1563 independent reflections
970 reflections with *I* > 2σ(*I*)
*R*_int_ = 0.040

#### Refinement

*R*[*F* ^2^ > 2σ(*F* ^2^)] = 0.044
*wR*(*F* ^2^) = 0.119
*S* = 0.98
1563 reflections
208 parameters
3 restraints
H-atom parameters constrained
Δρ_max_ = 0.11 e Å^−3^
Δρ_min_ = −0.13 e Å^−3^

### 

Data collection: *APEX2* (Bruker, 2005 ▶); cell refinement: *SAINT-Plus* (Bruker, 2004 ▶); data reduction: *SAINT-Plus* and *XPREP* (Bruker, 2004 ▶); program(s) used to solve structure: *SHELXS97* (Sheldrick, 2008 ▶); program(s) used to refine structure: *SHELXL97* (Sheldrick, 2008 ▶); molecular graphics: *ORTEP-3 for Windows* (Farrugia, 1997 ▶) and *DIAMOND* (Brandenburg, 1999 ▶); software used to prepare material for publication: *WinGX* (Farrugia, 1999 ▶) and *PLATON* (Spek, 2009 ▶).

## Supplementary Material

Crystal structure: contains datablock(s) global, I. DOI: 10.1107/S1600536812044443/cv5350sup1.cif

Structure factors: contains datablock(s) I. DOI: 10.1107/S1600536812044443/cv5350Isup2.hkl

Supplementary material file. DOI: 10.1107/S1600536812044443/cv5350Isup3.mol

Supplementary material file. DOI: 10.1107/S1600536812044443/cv5350Isup4.cml

Additional supplementary materials:  crystallographic information; 3D view; checkCIF report

## Figures and Tables

**Table 1:** Hydrogen-bond geometry (Å, °)

*D*—H⋯*A*	*D*—H	H⋯*A*	*D*⋯*A*	*D*—H⋯*A*
C3—H3⋯O1^i^	0.93	2.45	3.368 (5)	170

Symmetry code: (i) 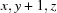.

**Fig. 1.**
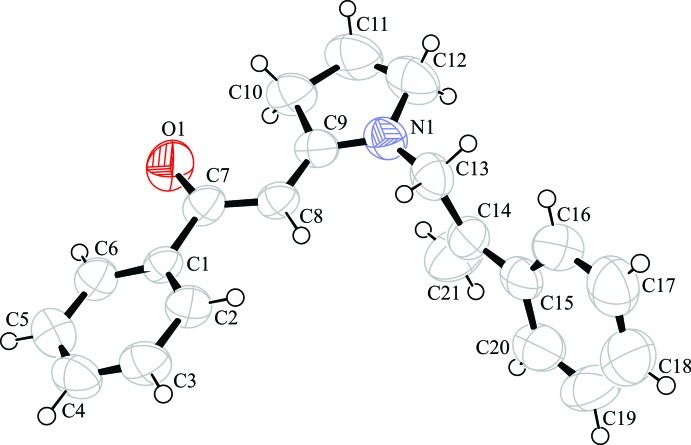
The molecular structure of (I) showing the atomic numbering scheme. Displacement ellipsoids are shown at the 50% probability level.

**Fig. 2.**
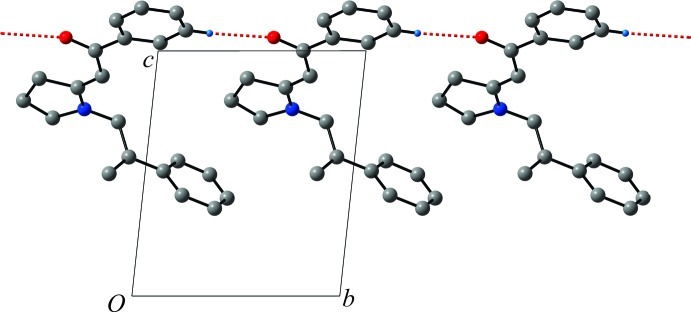
A portion of the crystal packing showing C—H···O hydrogen bonds as dashed red lines. H atoms not involved in hydrogen bonding are omitted for clarity.

## supplementary crystallographic information

### Comment

The title compound, (2*E*)-1-phenyl-2-[1-(2-phenylprop-2-en-1-yl)pyrrolidin-2-ylidene] ethanone, (I), was prepared as part of an ongoing project dealing with the use of enaminones as intermediates for alkaloid synthesis (Michael *et al.*, 1999).

In (I) (Fig. 1), two phenyl rings are twisted from the mean plane of the central pyrrolidine ring by 11.2 (1) and 67.3 (1)°, respectively. The (*E*) configuration and *s-cis* conformation of the exocyclic C═C bond of the enaminone are similar to those found in a related enaminone (Lemmerer *et al.*, 2007). In the crystal, weak intermolecular C—H···O hydrogen bonds (Table 1) link the molecules related by translation along the *b* axis into chains (Fig. 2).

### Experimental

The synthesis employed followed the Eschenmoser procedure (Roth *et al.*, 1971). A solution of phenacyl bromide (4.30 g, 21.6 mmol) and 1-(2-phenylprop-2-en-1-yl)pyrrolidine-2-thione (4.27 g, 19.6 mmol) in dry acetonitrile (20 ml) was stirred at room temperature under an argon atmosphere until precipitation of the adduct as a gum was complete. The mixture was briefly warmed to solubilize the precipitate, after which a solution of triphenylphosphine (5.66 g, 21.6 mmol) and triethylamine (3.31 ml, 23.6 mmol) in dry MeCN (20 ml) was added dropwise to induce extrusion of sulfur, and stirring was maintained for 18 h. The solvent was evaporated and the residue was taken up into ethyl acetate (200 ml) and washed with water (3 × 100 ml) and brine (50 ml). The organic phase was dried over MgSO_4_, filtered and evaporated to give an orange gum. Column chromatography on silica gel with hexane:ethyl acetate (3:2 *v/v*) afforded the title compound (5.42 g, 91%) as very pale yellow needles, m.p. 325—326 K.

### Refinement

The C-bound H atoms were geometrically positioned [C—H = 0.93 Å (alkenyl- and aromatic-H) and 0.97 Å (methylene-H)] and refined as riding with *U*_iso_(H) = 1.2*U*_eq_(C). In the absence of significant anomalous scatterers in the molecule, 1562 Friedel pairs were merged before the final refinement.

### Crystal data

**Table:**

C_21_H_21_NO	*Z* = 1
*M**_r_* = 303.39	*F*(000) = 162
Triclinic, *P*1	*D*_x_ = 1.188 Mg m^−^^3^
Hall symbol: P 1	Mo *K*α radiation, λ = 0.71073 Å
*a* = 5.7806 (6) Å	Cell parameters from 1287 reflections
*b* = 7.9407 (7) Å	θ = 2.2–24.8°
*c* = 9.6089 (9) Å	µ = 0.07 mm^−^^1^
α = 82.579 (7)°	*T* = 293 K
β = 76.793 (7)°	Prism, colourless
γ = 83.510 (7)°	0.4 × 0.2 × 0.19 mm
*V* = 424.21 (7) Å^3^	

### Data collection

**Table:**

Bruker APEXII CCD area-detector diffractometer	*R*_int_ = 0.040
ω scans	θ_max_ = 25.5°, θ_min_ = 2.2°
5222 measured reflections	*h* = −6→6
1563 independent reflections	*k* = −9→9
970 reflections with *I* > 2σ(*I*)	*l* = −11→11

### Refinement

**Table:**

Refinement on *F*^2^	3 restraints
Least-squares matrix: full	H-atom parameters constrained
*R*[*F*^2^ > 2σ(*F*^2^)] = 0.044	*w* = 1/[σ^2^(*F*_o_^2^) + (0.0653*P*)^2^] where *P* = (*F*_o_^2^ + 2*F*_c_^2^)/3
*wR*(*F*^2^) = 0.119	(Δ/σ)_max_ < 0.001
*S* = 0.98	Δρ_max_ = 0.11 e Å^−^^3^
1563 reflections	Δρ_min_ = −0.13 e Å^−^^3^
208 parameters	

### Special details

**Table:**

Geometry. All e.s.d.'s (except the e.s.d. in the dihedral angle between two l.s. planes) are estimated using the full covariance matrix. The cell e.s.d.'s are taken into account individually in the estimation of e.s.d.'s in distances, angles and torsion angles; correlations between e.s.d.'s in cell parameters are only used when they are defined by crystal symmetry. An approximate (isotropic) treatment of cell e.s.d.'s is used for estimating e.s.d.'s involving l.s. planes.

### Fractional atomic coordinates and isotropic or equivalent isotropic displacement parameters (Å^2^)

**Table:**

	*x*	*y*	*z*	*U*_iso_*/*U*_eq_	
C1	0.6399 (6)	0.8328 (4)	1.0527 (4)	0.0522 (9)	
C2	0.5691 (7)	1.0058 (4)	1.0341 (4)	0.0653 (11)	
H2	0.446	1.0433	0.9869	0.078*	
C3	0.6799 (8)	1.1226 (5)	1.0850 (5)	0.0752 (12)	
H3	0.6316	1.2382	1.0718	0.09*	
C4	0.8608 (8)	1.0687 (6)	1.1548 (5)	0.0785 (13)	
H4	0.9371	1.1472	1.1881	0.094*	
C5	0.9283 (7)	0.8981 (6)	1.1751 (5)	0.0789 (13)	
H5	1.0501	0.8604	1.2233	0.095*	
C6	0.8181 (7)	0.7835 (5)	1.1250 (4)	0.0659 (11)	
H6	0.8656	0.668	1.1406	0.079*	
C7	0.5255 (7)	0.6979 (4)	1.0017 (4)	0.0636 (11)	
C8	0.3853 (7)	0.7440 (4)	0.8977 (4)	0.0585 (10)	
H8	0.3737	0.8574	0.8591	0.07*	
C9	0.2666 (7)	0.6322 (5)	0.8508 (4)	0.0636 (11)	
C10	0.2782 (9)	0.4424 (5)	0.8878 (5)	0.0838 (13)	
H10A	0.2124	0.4137	0.9893	0.101*	
H10B	0.4419	0.3929	0.8653	0.101*	
C11	0.1326 (11)	0.3781 (7)	0.7980 (7)	0.1127 (19)	
H11A	0.2347	0.3109	0.7267	0.135*	
H11B	0.0148	0.3068	0.8582	0.135*	
C12	0.0139 (10)	0.5276 (7)	0.7269 (6)	0.1016 (17)	
H12A	−0.1575	0.5333	0.7633	0.122*	
H12B	0.0477	0.5234	0.6238	0.122*	
C13	0.0360 (8)	0.8452 (6)	0.7109 (4)	0.0773 (12)	
H13A	0.0421	0.9211	0.7811	0.093*	
H13B	−0.1294	0.8469	0.7046	0.093*	
C14	0.1767 (8)	0.9137 (6)	0.5674 (4)	0.0778 (12)	
C15	0.0892 (8)	1.0875 (6)	0.5101 (4)	0.0751 (12)	
C16	−0.1450 (9)	1.1520 (6)	0.5513 (6)	0.0909 (14)	
H16	−0.2506	1.0866	0.6185	0.109*	
C17	−0.2279 (11)	1.3117 (8)	0.4954 (7)	0.1099 (17)	
H17	−0.3873	1.3518	0.5237	0.132*	
C18	−0.0743 (16)	1.4078 (8)	0.3995 (7)	0.1170 (19)	
H18	−0.1294	1.5144	0.3614	0.14*	
C19	0.1563 (17)	1.3526 (10)	0.3579 (6)	0.121 (2)	
H19	0.2612	1.422	0.2942	0.145*	
C20	0.2381 (9)	1.1892 (8)	0.4114 (5)	0.1007 (17)	
H20	0.3965	1.1492	0.3792	0.121*	
C21	0.3663 (10)	0.8275 (8)	0.4994 (6)	0.1161 (19)	
H21A	0.4527	0.8741	0.4117	0.139*	
H21B	0.4147	0.7197	0.5389	0.139*	
O1	0.5650 (7)	0.5495 (3)	1.0546 (4)	0.0998 (11)	
N1	0.1148 (6)	0.6753 (4)	0.7626 (3)	0.0725 (10)	

### Atomic displacement parameters (Å^2^)

**Table:**

	*U*^11^	*U*^22^	*U*^33^	*U*^12^	*U*^13^	*U*^23^
C1	0.067 (2)	0.0394 (18)	0.049 (2)	−0.0063 (17)	−0.0088 (18)	−0.0052 (16)
C2	0.081 (3)	0.041 (2)	0.076 (3)	−0.004 (2)	−0.019 (2)	−0.0123 (18)
C3	0.094 (3)	0.048 (2)	0.086 (3)	−0.011 (2)	−0.016 (3)	−0.018 (2)
C4	0.086 (3)	0.078 (3)	0.077 (3)	−0.024 (3)	−0.012 (3)	−0.027 (2)
C5	0.074 (3)	0.089 (3)	0.079 (3)	−0.004 (3)	−0.022 (2)	−0.019 (3)
C6	0.080 (3)	0.052 (2)	0.065 (3)	−0.003 (2)	−0.015 (2)	−0.0099 (19)
C7	0.089 (3)	0.044 (2)	0.058 (3)	−0.007 (2)	−0.012 (2)	−0.0082 (19)
C8	0.074 (2)	0.044 (2)	0.057 (2)	−0.0108 (19)	−0.009 (2)	−0.0080 (18)
C9	0.074 (3)	0.063 (2)	0.054 (2)	−0.017 (2)	−0.002 (2)	−0.017 (2)
C10	0.111 (3)	0.058 (2)	0.088 (3)	−0.024 (2)	−0.015 (3)	−0.023 (2)
C11	0.135 (5)	0.097 (4)	0.118 (5)	−0.047 (4)	−0.020 (4)	−0.034 (4)
C12	0.107 (4)	0.113 (4)	0.097 (4)	−0.035 (3)	−0.019 (3)	−0.043 (3)
C13	0.069 (3)	0.101 (3)	0.065 (3)	−0.009 (2)	−0.015 (2)	−0.017 (2)
C14	0.072 (3)	0.106 (3)	0.055 (2)	−0.022 (2)	−0.011 (2)	−0.001 (2)
C15	0.077 (3)	0.108 (3)	0.048 (2)	−0.032 (3)	−0.015 (2)	−0.014 (2)
C16	0.091 (4)	0.091 (3)	0.094 (3)	−0.023 (3)	−0.022 (3)	−0.004 (3)
C17	0.125 (5)	0.108 (4)	0.112 (5)	−0.010 (4)	−0.052 (4)	−0.021 (4)
C18	0.172 (7)	0.113 (5)	0.083 (4)	−0.042 (5)	−0.049 (4)	−0.009 (4)
C19	0.179 (7)	0.126 (5)	0.064 (4)	−0.078 (5)	−0.019 (4)	0.010 (3)
C20	0.107 (4)	0.145 (5)	0.055 (3)	−0.051 (4)	−0.005 (3)	−0.015 (3)
C21	0.108 (4)	0.129 (4)	0.085 (3)	0.000 (3)	0.025 (3)	−0.007 (3)
O1	0.165 (3)	0.0361 (15)	0.114 (3)	−0.0150 (15)	−0.065 (2)	0.0006 (14)
N1	0.082 (2)	0.075 (2)	0.067 (2)	−0.0187 (19)	−0.018 (2)	−0.0181 (18)

### Geometric parameters (Å, º)

**Table:**

C1—C6	1.365 (5)	C11—H11B	0.97
C1—C2	1.389 (4)	C12—N1	1.478 (6)
C1—C7	1.507 (5)	C12—H12A	0.97
C2—C3	1.380 (5)	C12—H12B	0.97
C2—H2	0.93	C13—N1	1.437 (5)
C3—C4	1.368 (6)	C13—C14	1.501 (6)
C3—H3	0.93	C13—H13A	0.97
C4—C5	1.368 (6)	C13—H13B	0.97
C4—H4	0.93	C14—C21	1.309 (6)
C5—C6	1.360 (6)	C14—C15	1.495 (6)
C5—H5	0.93	C15—C20	1.371 (6)
C6—H6	0.93	C15—C16	1.379 (6)
C7—O1	1.239 (4)	C16—C17	1.390 (7)
C7—C8	1.413 (5)	C16—H16	0.93
C8—C9	1.358 (5)	C17—C18	1.348 (8)
C8—H8	0.93	C17—H17	0.93
C9—N1	1.342 (5)	C18—C19	1.341 (8)
C9—C10	1.500 (5)	C18—H18	0.93
C10—C11	1.503 (7)	C19—C20	1.404 (8)
C10—H10A	0.97	C19—H19	0.93
C10—H10B	0.97	C20—H20	0.93
C11—C12	1.472 (7)	C21—H21A	0.93
C11—H11A	0.97	C21—H21B	0.93
			
C6—C1—C2	117.7 (3)	C11—C12—N1	104.6 (4)
C6—C1—C7	118.8 (3)	C11—C12—H12A	110.8
C2—C1—C7	123.5 (3)	N1—C12—H12A	110.8
C3—C2—C1	120.5 (4)	C11—C12—H12B	110.8
C3—C2—H2	119.7	N1—C12—H12B	110.8
C1—C2—H2	119.7	H12A—C12—H12B	108.9
C4—C3—C2	120.2 (4)	N1—C13—C14	115.3 (4)
C4—C3—H3	119.9	N1—C13—H13A	108.5
C2—C3—H3	119.9	C14—C13—H13A	108.5
C3—C4—C5	119.4 (4)	N1—C13—H13B	108.5
C3—C4—H4	120.3	C14—C13—H13B	108.5
C5—C4—H4	120.3	H13A—C13—H13B	107.5
C6—C5—C4	120.2 (4)	C21—C14—C15	122.8 (4)
C6—C5—H5	119.9	C21—C14—C13	121.7 (5)
C4—C5—H5	119.9	C15—C14—C13	115.5 (4)
C5—C6—C1	122.0 (4)	C20—C15—C16	116.7 (5)
C5—C6—H6	119	C20—C15—C14	121.3 (4)
C1—C6—H6	119	C16—C15—C14	122.0 (4)
O1—C7—C8	123.9 (3)	C15—C16—C17	122.0 (5)
O1—C7—C1	116.2 (4)	C15—C16—H16	119
C8—C7—C1	119.9 (3)	C17—C16—H16	119
C9—C8—C7	123.8 (3)	C18—C17—C16	119.1 (6)
C9—C8—H8	118.1	C18—C17—H17	120.5
C7—C8—H8	118.1	C16—C17—H17	120.5
N1—C9—C8	124.8 (3)	C19—C18—C17	121.3 (6)
N1—C9—C10	108.2 (3)	C19—C18—H18	119.3
C8—C9—C10	127.0 (4)	C17—C18—H18	119.3
C9—C10—C11	105.7 (4)	C18—C19—C20	119.5 (6)
C9—C10—H10A	110.6	C18—C19—H19	120.3
C11—C10—H10A	110.6	C20—C19—H19	120.3
C9—C10—H10B	110.6	C15—C20—C19	121.3 (6)
C11—C10—H10B	110.6	C15—C20—H20	119.4
H10A—C10—H10B	108.7	C19—C20—H20	119.4
C12—C11—C10	107.5 (4)	C14—C21—H21A	120
C12—C11—H11A	110.2	C14—C21—H21B	120
C10—C11—H11A	110.2	H21A—C21—H21B	120
C12—C11—H11B	110.2	C9—N1—C13	126.6 (3)
C10—C11—H11B	110.2	C9—N1—C12	113.4 (4)
H11A—C11—H11B	108.5	C13—N1—C12	119.8 (4)
			
C6—C1—C2—C3	1.3 (5)	N1—C13—C14—C15	177.5 (3)
C7—C1—C2—C3	179.2 (4)	C21—C14—C15—C20	−21.8 (7)
C1—C2—C3—C4	−0.2 (6)	C13—C14—C15—C20	156.6 (4)
C2—C3—C4—C5	−0.8 (6)	C21—C14—C15—C16	156.8 (5)
C3—C4—C5—C6	0.6 (7)	C13—C14—C15—C16	−24.7 (5)
C4—C5—C6—C1	0.5 (6)	C20—C15—C16—C17	0.4 (6)
C2—C1—C6—C5	−1.5 (6)	C14—C15—C16—C17	−178.3 (4)
C7—C1—C6—C5	−179.5 (4)	C15—C16—C17—C18	−1.0 (7)
C6—C1—C7—O1	14.8 (5)	C16—C17—C18—C19	−0.4 (8)
C2—C1—C7—O1	−163.2 (4)	C17—C18—C19—C20	2.2 (8)
C6—C1—C7—C8	−164.4 (4)	C16—C15—C20—C19	1.5 (6)
C2—C1—C7—C8	17.7 (5)	C14—C15—C20—C19	−179.8 (4)
O1—C7—C8—C9	4.2 (6)	C18—C19—C20—C15	−2.8 (8)
C1—C7—C8—C9	−176.7 (3)	C8—C9—N1—C13	−5.1 (6)
C7—C8—C9—N1	173.4 (4)	C10—C9—N1—C13	174.4 (4)
C7—C8—C9—C10	−6.0 (6)	C8—C9—N1—C12	179.0 (4)
N1—C9—C10—C11	5.6 (5)	C10—C9—N1—C12	−1.5 (5)
C8—C9—C10—C11	−174.9 (4)	C14—C13—N1—C9	94.1 (4)
C9—C10—C11—C12	−7.5 (5)	C14—C13—N1—C12	−90.3 (5)
C10—C11—C12—N1	6.6 (5)	C11—C12—N1—C9	−3.3 (5)
N1—C13—C14—C21	−4.0 (6)	C11—C12—N1—C13	−179.5 (4)

### Hydrogen-bond geometry (Å, º)

**Table:**

*D*—H···*A*	*D*—H	H···*A*	*D*···*A*	*D*—H···*A*
C3—H3···O1^i^	0.93	2.45	3.368 (5)	170

Symmetry code: (i) *x*, *y*+1, *z*.

## References

[bb1] Brandenburg, K. (1999). *DIAMOND* Crystal Impact GbR, Bonn, Germany.

[bb2] Bruker (2004). *SAINT-Plus* and *XPREP* Bruker AXS Inc., Madison, Wisconsin, USA.

[bb3] Bruker (2005). *APEX2* Bruker AXS Inc., Madison, Wisconsin, USA.

[bb4] Farrugia, L. J. (1997). *J. Appl. Cryst.* **30**, 565.

[bb5] Farrugia, L. J. (1999). *J. Appl. Cryst.* **32**, 837–838.

[bb6] Lemmerer, A., Michael, J. P., Pienaar, D. P. & Sannasy, D. (2007). *Acta Cryst.* E**63**, o98–o99.

[bb7] Michael, J. P., de Koning, C. B., Gravestock, D., Hosken, G. D., Howard, A. S., Jungmann, C. M., Krause, R. W. M., Parsons, A. S., Pelly, S. C. & Stanbury, T. V. (1999). *Pure Appl. Chem.* **71**, 979–988.

[bb8] Roth, M., Dubs, P., Götschi, E. & Eschenmoser, A. (1971). *Helv. Chim. Acta*, **54**, 710–734.

[bb9] Sheldrick, G. M. (2008). *Acta Cryst.* A**64**, 112–122. 10.1107/S0108767307043930 18156677

[bb10] Spek, A. L. (2009). *Acta Cryst.* D**65**, 148–155. 10.1107/S090744490804362X PMC2631630 19171970

